# Next‐Generation Sequencing: An Advanced Diagnostic Tool for Detection of Pancreatic Disease/Disorder

**DOI:** 10.1002/jgh3.70061

**Published:** 2024-11-27

**Authors:** Suvro Biswas, Shamima Afrose, Mohasana Akter Mita, Md. Robiul Hasan, Mst. Sharmin Sultana Shimu, Shahriar Zaman, Md. Abu Saleh

**Affiliations:** ^1^ Miocrobiology Laboratory, Department of Genetic Engineering and Biotechnology University of Rajshahi Bangladesh; ^2^ Department of Genetic Engineering and Biotechnology University of Rajshahi Rajshahi Bangladesh

**Keywords:** IPMN, LAPC, MCN, NGS, pancreatic disease, pancreatitis, PDAC

## Abstract

The pancreas is involved in digestion and glucose regulation in the human body. Given the recognized link between chronic pancreatitis and pancreatic cancer, addressing pancreatic disorders and pancreatic cancer is particularly challenging. This review aims to highlight the limitations of traditional methods in diagnosing pancreatic disorders and cancer and explore several next‐generation sequencing (NGS) approaches as a promising alternative. There are distinct clinical symptoms that are shared by a number of clinical phenotypes of pancreatic illness induced by particular genetic mutations. Traditional diagnostic methods encompass computed tomography, magnetic resonance imaging, contrast‐enhanced Doppler ultrasound, endoscopic ultrasound, endoscopic retrograde cholangiopancreatography, transabdominal ultrasound, laparoscopy, and positron emission tomography have a prognostic ability of only 5% or less and a 5‐year survival rate. Genetic sequencing can be employed as an alternative to conventional diagnostic techniques. Sanger sequencing and NGS are currently largely operated genome analysis, with no exception for pancreatic disease diagnosis. The NGS methods can sequence millions to billions of short DNA fragments, enabling enormous sample screening in a short amount of time with low‐abundance detection, like in 0.1%–1% mutation prevalence declining approximate cost. Whole‐genome sequencing, whole‐exome sequencing, RNA sequencing, and single‐cell NGS are a few NGS methods utilized to diagnose pancreatic disease. For both research and clinical applications, the NGS techniques can provide a precise diagnosis of pancreatic disorders in a short amount of time at a reasonable expenditure.

## 1 Introduction to Pancreas and Pancreatic Disorders

The pancreas is an expanded, tender, plain, lobulated yellow‐colored gland, and remains on the back of the abdominal wall. It is located in a retroperitoneal space and is made up of a thin capsule. The pancreas has a hammer or hook‐like shape [1]. The name “pancreas” derived from the Greek words “pan” (all) and “creas” (flesh). It weighs between 70 and 150 g and has an average volume of 72 cm^3^ in humans. Its length, height, and width are 12–20, 3–5, and 1–3 cm, respectively [2, 3]. The pancreas is elongated and has a form similar to a hammer or a hook. In humans, the pancreas is microscopically divided into The head, neck, body, and tail of the pancreas in humans are used to identify the region of the organ from proximal to distal [2, 3]. The tail continues to be to the left of the midline, while the head and neck continue to be somewhat to the right of the midline. The body goes to the left and slightly slopes upward to become continuous with the tail [1]. The pancreas is a secondary retroperitoneal organ that is located on the posterior wall of the abdominal cavity and is enclosed in a thin capsule. The pancreatic duct, also known as the “duct of Wirsung,” starts in the organ's tail, travels throughout it, and then joins the common bile duct. Hepatopancreatic ampulla, also known as the “ampulla of Vater,” is the name of this union, which is found on the main duodenal papilla [2, 4]. Two buds for the pancreas grow on the ventral and dorsal sides of the duodenum, respectively. The dorsal bud develops on the opposite side of the gut tube from the ventral bud, which grows immediately next to the hepatic diverticulum [3].

The pancreas is a complex gland made up of exocrine and endocrine cells, two very different types of glandular tissue. It is an essential organ for life because it is crucial for digestion and glucose regulation. 95%–99% of the mature pancreas is made up of exocrine components, which mainly secrete digestive enzymes into the intestine. A branching, acinar, and lobulated gland makes up the exocrine component of the pancreas. The pyramidal, basal‐nucleated acini in which the secretory cells are arranged. The islets of Langerhans, which are dense spheroidal clusters dotted throughout the exocrine tissue, make up the endocrine pancreas. There are four main types of endocrine cells in the endocrine pancreas. Insulin is secreted from the β (or B) cells, moreover, amylin is an insulin antagonist. Furthermore, the α (or A or A2) cells, δ (or D or A1) cells, and the PP (or F) cells secrete glucagon, somatostatin (SS), and pancreatic polypeptide (PP), respectively [3, 5].

The retroperitoneal region of the exocrine pancreas, which is difficult to access, is where the majority of people's exocrine pancreas is located. Since benign disorders are rare and the pancreas has a large reserve of receptivity, pancreatic insufficiency signs and symptoms only become obvious when more than 90% of the pancreas is not functioning. However, there are also extremely deadly pancreatic illnesses, such as “acute pancreatitis,” which has a high fatality rate [6, 7, 8]. Many individuals who are diagnosed with “pancreatic cancer” may never be cured, and “chronic pancreatitis” causes a great deal of misery and is extremely difficult to treat. Patients with cystic fibrosis may potentially experience pancreatic abnormalities in addition to these illnesses. Additionally, mutations can also be the cause of several pancreatitis kinds, including, “hereditary pancreatitis,” “recurrent pancreatitis,” “pancreatic ductal adenocarcinoma,” “locally advanced pancreatic carcinoma,” “intraductal papillary mucinous neoplasms,” and so forth. Additionally, a connection between diabetes and pancreatic cancer exists but is difficult to demonstrate, and a connection between “chronic pancreatitis” and “pancreatic cancer” has also been confirmed [8, 9, 10, 11, 12].

## 2 Clinical Features of Pancreatic Disorders

“Acute pancreatitis” or “hereditary pancreatitis” cause severe upper abdominal pain. Back discomfort that radiates, along with jaundice, nausea, fever, and vomiting, are all possible correlations. In the most severe situations, this reaction could result in several organs failing and early death. Acute pancreatitis in older people should also be treated in an ICU or transitional care facility [8, 13, 14, 15]. Up to 85% of individuals with “chronic pancreatitis” or “recurrent pancreatitis” experience pain, which is typically the most common symptom. Steatorrhea is a symptom of exocrine deficiency, which can also lead to malnutrition, weight loss, and inadequate levels of fat‐soluble vitamins. In addition, type 3C diabetes mellitus is a disease brought on by an endocrine deficiency [16, 17, 18] (Table 1).

**TABLE 1:** Phenotype, their mutations, and common clinical features of different pancreatic diseases.

Clinical phenotype	Genetic mutation	Most common clinical features
Acute pancreatitis or hereditary pancreatitis [8]	V39E and N42S mutations in cationic trypsinogen gene (PRSS1) [19]	Fever, hypovolemia, tachypnea, tachycardia, or hypoxia as a phenomenon of early inflammatory reaction [13]
Chronic pancreatitis or recurrent pancreatitis [8]	N34S, V46D, and N34S mutations in serine protease inhibitor Kazal type 1 gene (SPINK1) [19]	Exocrine pancreatic deficiency, abdominal pain, and diabetes [16]
Pancreatic cancer	Mutations occur in p16/CDKN2A, SMAD4/DPC4, KRAS2, and TP53 gene [20]	Jaundice, back pain, abdominal pain, unintentional weight loss, pancreatitis, loss of hunger, etc. [21]
Pancreatic ductal adenocarcinoma	Mutations arise in BRCA1/2, KDM6A, APOBEC, KRAS, CDKN2A/p16, TP53, SMAD4/DPC4 in tandem with RNF43 [22, 23, 24, 25]	Jaundice, abdominal or back pain, pruritus, weight loss, and vomiting or nausea [26]
Locally advanced pancreatic carcinoma	Mutations occur in KRAS gene such as KRAS G12V mutation in conjunction with CDKN2A, NOTCH1/2, SMAD4/DPC4, and TP53 [27, 28, 29, 30]	Abnormal liver function, abdominal pain, jaundice, dyspepsia, new‐onset diabetes, vomiting or nausea, weight loss, and back pain [27]
Intraductal papillary mucinous neoplasms	Mutations happen in KRAS, GNAS, RNF43, BRAF, CDKN2A, PIK3CA, STK11, CTNNB1, APC, ATM, TP53, PTCH1, SUFU, and KLF4 [31, 32, 33]	Abdominal symptoms including epigastric discomfort or pain, backache, long‐standing hyperamylasemia, weight loss, diabetes, jaundice, and steatorrhea [34]
Pancreatic cysts	R201H, and R844H mutation of GNAS gene; G12V, G12D, G12L, Q61R, G13D, Q61H, and D153V mutation regarding KRAS gene; VHL P91S; TP53 R248Q [35]	Jaundice, abdominal or back pain, steatorrhea, a palpable mass, and unexplained weight loss [36]
Mucinous cystic neoplasms	Mutations occur in PIK3CA E545K (G1633A), AKT1/PKB, KRAS, TP53, p16, SMAD4/DPC4, RNF43, and P16INK4A/CDKN2A [37, 38]	Abdominal pain, nausea or vomiting, weight loss, back pain, epigastric fullness, and heaviness [39, 40]

Obstructive jaundice, which is brought on by condensation of the bile duct at the pancreas head, affects the majority of patients with “pancreatic cancer.” Weight loss, epigastric or back pain, and hazy stomach sensations are further signs of pancreatic cancer [41, 42]. Though chronic pancreatitis is extremely uncommon and accounts for fewer than 5% of all occurrences of pancreatic cancer, several studies have confirmed a link between the two conditions. Although it is difficult to prove a connection, long‐term diabetes may increase the risk of pancreatic cancer by 50% [8, 9, 10, 11, 43].

Additionally, the emergence of several molecular abnormalities and genetic alterations indicating late clinical manifestation from the illness onset contributes to the rise of PDAC or pancreatic ductal adenocarcinoma over decades [44]. However, the invasive foci measured in the sub‐centimeter range in the early cases of PDAC with the ability to metastasis contain a greater risk of recurrence. In addition to 51% weight loss, 39% abdominal pain, 13% nausea or vomiting, and 11% pruritus, almost 75% of PDAC patients also have jaundice [45]. However, during the course of the illness, either newly developing diabetes or complications from preexisting diabetes occur. Dark urine, pruritus, and acholic stools are further signs of conjugated hyperbilirubinemia brought on by the occlusion of the common bile duct [26, 46].

Additionally, the location of the tumor within the pancreas affects the clinical characteristics of LAPC, or locally advanced pancreatic carcinoma. About 60%–70% of cases of pancreatic tumors that originate in the pancreatic neck or head result in biliary blockage and painless jaundice [47]. The hepatic, celiac, and primary mesenteric veins, together with the portal vein, are also disrupted by tumors that arise from the pancreatic body, which results in back pain. However, rarer anatomical features that indicate to be progressed during diagnosis allow pancreatic tail tumors to evolve. Additionally, malignant obstruction of the pancreatic duct results in pancreatic enzyme insufficiency, which leads to malabsorption of fat and postprandial abdominal pain, loose stools, flatulence, and possibly steatorrhea [27].

In addition, partial or complete occlusion concerning the predominant pancreatic duct harboring viscid mucin generates abdominal symptoms including jaundice, epigastric pain or discomfort, weight loss, and backache, in addition to long‐standing hyperamylasemia in 70%–80% of cases of IPMNs or intraductal papillary mucinous neoplasms [48, 49, 50]. Likewise, chronic persistent blockage, which causes diabetes, steatorrhea, and/or pancreatic insufficiency. However, when the disease progresses, malignant IPMN together with common bile duct or ampulla involvement through mural nodules as a result of viscid mucin eroding the ampulla and the significant common bile duct constriction results in jaundice. Patients with IPMNs also exhibit mild to moderately severe acute pancreatitis [51].

About 70% of pancreatic cyst patients have no symptoms, although others develop jaundice, unexplained weight loss, back or abdominal pain, a palpable lump, or steatorrhea over time [52, 53, 54]. Similarly, back pain (7%–40%), nausea or vomiting (20%–30%), an abdominal mass (30%–60%), and epigastric fullness and heaviness (60%–90%) describe the clinical appearance of mucinous cystic neoplasms [55, 56]. However, the majority of MCNs are fundamentally asymptomatic and slow growing [39, 57].

## 3 Traditional Diagnostic Approach for Pancreatic Disease/Tumor/Cancer

**FIGURE 1:**
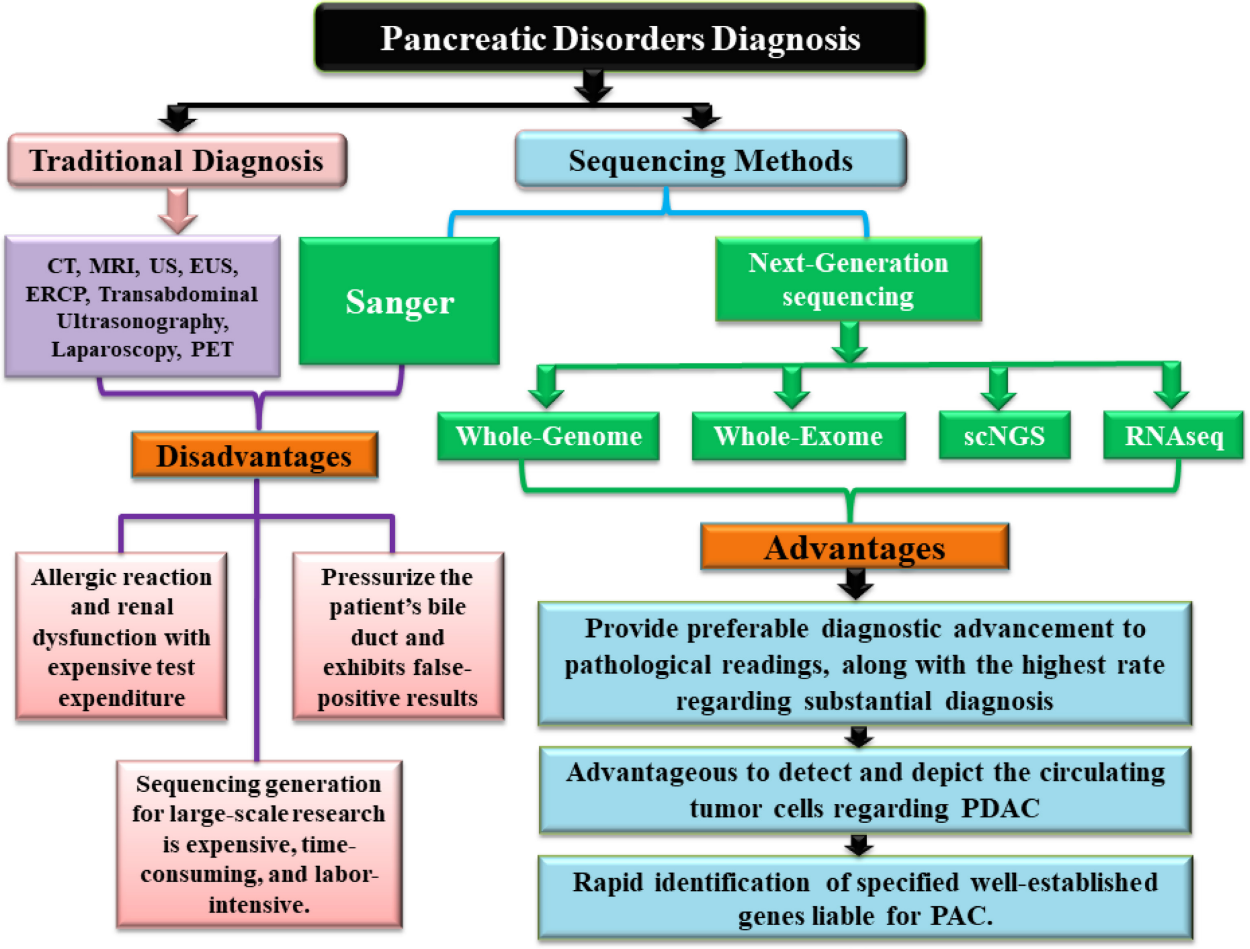
Diagrammatic depictions of traditional and next‐generation sequencing for detecting pancreatic disease/disorder.

Pancreatic cancer become one of the internecine highly malignancies worldwide with darkling prognosticate ability of only near about 5% along with 5 years survival ratio. However, several significant diagnostic approaches to increase preoperative staging and pancreatic management facilities are available including computed tomography (CT), magnetic resonance imaging (MRI), contrast‐enhanced Doppler ultrasound (US), endoscopic ultrasound (EUS), endoscopic retrograde cholangiopancreatography (ERCP), transabdominal ultrasonography, laparoscopy and positron emission tomography (PET) (Figure 1) [55, 56, 57, 58].

CT is known for its elementary expression scheme in suspected pancreatic carcinoma. This technique has evolved over time from conventional CT to helical or spiral CT and finally to new multi‐detector spiral CT. This method of detection exhibits rapid rotational identification, allowing for three‐dimensional realignment and high‐yielding resolution of pancreatic cancer in its early stages. The CT scans are often performed with less than 300 mg I/mL intravenous contrast at injection rates of 3–5 mL/s, with scans obtained in two phases: pancreatic parenchymal phase and portal venous phase. According to several experimental results, CT has a detection sensitivity range of about 76%–96% [59, 60, 61, 62, 63, 64, 65].

As a second‐line imaging modality for detection, MRI, a technique, is a staging and diagnostic process that shows advanced soft tissue contrast in comparison to CT. Pancreatic carcinoma or cancer can be shown on MRI as hypointense T1‐weighted images and isointense or hyperintense T2‐weighted images. MR imaging depicts the tumor as resisted enhancement in the early stage but expresses continuous expansion in the latter stage. It is interesting that MRI has a detection rate of roughly 83%–95%, which is very similar to CT. Pancreatic cancers that are discovered to be hypovascularized are diagnosed using contrast‐enhanced Doppler US which is based on coded technology and an amalgam of phase inversion harmonics. According to a report, this helpful procedure has a 90%–95% specificity and sensitivity for detecting cancer [62, 66, 67, 68, 69, 70, 71].

On the other hand, EUS, which uses high‐frequency prominent tiny US transducers, can examine the typical characteristics of pancreatic cancer. This wave is able to capture abundant resolution imaging of arrangements that are smaller than 1 mm in size and allows for the accurate evaluation of pancreatic lesions that are seen from various angles. This diagnostic method has demonstrated detection sensitivity above 90%. The ability to scan the biliary channels and pancreatic structure, which have historically been incomparable to destinations, makes ERCP a wonderful tool for identifying pancreatic malignancy in patients [62, 66, 72, 73, 74, 75].

Transabdominal ultrasonography is a simple and reliable method that is utilized as the first imaging test for patients who may have pancreatic problems. Transabdominal US essentially offers noninvasive access to the pancreaticobiliary system without subjecting patients to radiation exposure while confirming the presence of cholecystitis or cholelithiasis and disclosing benign etiologies. Additionally, this method increases pancreatic cancer detection sensitivity from 50% to 90%. However, laparoscopy indicates a very good chance that pancreatic cancer would spread, eventually reaching the peritoneum and overtaking diagnostic tools. After unnecessary surgery, a number of problems that are difficult to treat may arise, but a laparoscopy can simply be avoided [62, 76, 77, 78, 79]. PET shows its detecting feature as a noninvasive differentiation to image using a glucose analog entitle 18F‐fluoro‐2‐deoxy‐D‐glucose (FDG) that is radiotracer labeled which is acting as aggregating improved glycolytic metabolism in the cell. The PET has high sensitivity from 84.4% to 96.8% identifying pancreatic malignancy reported by several experimental studies [58, 60, 72, 77, 78].

## 4 Limitations Associated With Traditional Diagnostic Method

Nearly all‐diagnostic techniques for detecting pancreatic cancer encounter a number of difficulties. It is challenging to detect isoattenuating tumors in CT that are less than 20 mm. The majority of research revealed that 11% of tumors are isoattenuating in the pancreatic cancer detection process, which is crucial information. Instead of employing an MRI to explore tumor staging and pancreatic diagnosis, this method contrasts allergic reaction and renal dysfunction in patients while also costing them a lot of money for tests [62, 63, 66, 80, 81].

The existence of repressed overspreading intestinal gas and a lack of propeller's skill effect on contrast‐enhanced Doppler ultrasonography (US) sensitivity in pancreatic lesions are the two main causes for concern. On the other hand, EUS is a highly sought‐after skill for technical operators despite being an intrusive process that poses high‐risk issues for visualizing cross‐sectional imaging. Conversely, diagnostic methods utilizing ERCP procedures compressed the patient's bile duct and displayed reduced clinical sensitivity during diagnosis [62, 66, 82, 83, 84, 85].

The diagnostic staging scheme for transabdominal US has various limitations since close to 15%–20% of the time the test results are unsatisfactory because of prior surgery, being obese, or having intestinal gas obstruction, and this method is strongly advised for qualified operators. Furthermore, the structure of small primary tumors and extended cancers in regional lymph nodes are challenging to detect using this method. Contrarily, people who have laparoscopy and are later diagnosed with it must need surgical resection [62, 86, 87, 88, 89]. In PET, during the measurement of locoregional involvement, this method shows lacking spatial resolution, which is a crucial step in cancer staging. Behind this, it uses FDG which is accumulated in acute and chronic pancreatitis and exhibits false‐positive interpreting results on PET imaging [63, 66, 75].

## 5 Sequencing as an Alternative to the Traditional Pancreatic Disease Diagnosis Approach

Nowadays, genome analysis is predominantly conducted by not only Sanger sequencing but also next‐generation sequencing (NGS), with no exception for pancreatic disease diagnosis. Even though Sanger sequencing is comparatively uncomplicated and straightforward, being the gold standard of DNA sequencing, the approach is expensive, in tandem with time and labor‐intensive for large‐scale sequencing generation [90, 91, 92]. On the contrary, the NGS technique can sequence millions to billions of short DNA fragments enabling massive sample screening within a short period that can exhibit low‐abundance detection, such as in 0.1%–1% mutation prevalence lowering approximate cost [93, 94, 95, 96, 97].

## 6 Several NGS Approaches for Pancreatic Disease Diagnosis

### 6.1 Whole‐Genome Sequencing (WGS)

A contemporary investigation was the first to report WGS or whole‐genome sequencing, spotting approximately 132 billion mappable bases and recognizing 142 somatic coding cases incorporating insertion or deletions, point mutations, in tandem with chromosomal CNVs, which stands for “copy number variants” excluding translocation among three distinct PAC patients. The WGS specified several well‐established genes liable for PAC, including SMAD4, KRAS, MYC, TP53, BRCA2, and CDKN2A (p16) in addition to a contribution of DNA repair genes influenced by the somatic event's absence that reveals their contribution toward patient's current situation [98]. Another WGS of 638 FPC patients based on the germline DNA was conducted to analyze an explicit FPC genetic basis referring to the inherited pancreatic cancer as highly heterogeneous and reporting susceptibility genes elevating pancreatic cancer risk including, CDKN2A, BUB1B, FANCC, ATM, CPA1, BRCA2, FANCG, and PALB2 [94].

### 6.2 Whole‐Exome Sequencing (WES)

Whole‐exome sequencing, abbreviated as WES, an NGS strategy, regarded to be an alternative for a molecular diagnosis regarding PDAC (pancreatic ductal adenocarcinoma) cases, can provide preferable diagnostic advancement to pathological readings, along with the highest rate regarding substantial diagnosis and descending dropout rate before investigation [95]. Therefore, an improved diagnosis regarding LAPC (locally advanced pancreatic carcinoma) is achievable as 93.75% of cases are analyzed by NGS that provide adequately profound coverage and recognize pathogenic variants skipped through traditional sequencing approaches in a significantly tiny portion of the tumor defending, the requisition of the NGS with definitive diagnostic modalities. Nonetheless, targeted resequencing of specific genes comprehended to be mutated in pancreatic cancer provides a time‐ and cost‐effective manner for PDAC molecular diagnosis [96]. As PDACs frequently consist of a reasonable amount of nonneoplastic cells, WES is utilized to enhance sensitivity regarding somatic mutation detection that noticed 1409 somatic mutations typically in KRAS (84.6%) and TP53 (71.8%) in conjunction with 33.3% and 12.8% in SMAD4 and CDKN2A, respectively [99]. Another WES of solid pancreatic tumors mainly PDAC, flinching from fine‐needle aspiration (FNA) material, witnessed KRAS (93.7%), TP53 (56.0%), and CDKN2A (50.0%) as more frequent alterations [97, 98].

### 6.3 RNA Sequencing (RNA‐Seq)

The first exhaustive transcriptome investigation utilizing RNA‐seq in diagnosing pancreatic cancer recognized about 2736 DEGs (differentially expressed genes) retaining a lower than 0.05 false discovery rate that incorporates 1554 upregulated and 1182 downregulated, along with six microRNAs such as miR‐27b, miR‐4451, miR‐614, miR‐217, miR‐612, and miR‐3609. The study was validated by overexpression of HOXA10, SERPINB5, SI, CDX1, in conjunction with KRT16 genes in 20 supplementary tissues by RT‐PCR approach, in tandem with KRT16 overexpression affirmed through protein level investigation [99]. In addition, RNA sequencing yielded more than 100 million mapped reads, in conjunction with 1841 and 1939 genes with significant expression transitions for two patients observed in the tumor [93].

### 6.4 Single‐Cell Next‐Generation Sequencing (scNGS)

Relying on WGA or whole‐genome amplification, the scNGS abbreviated from single‐cell NGS is considered to be an advantageous approach to detect and depict the CTC or circulating tumor cells, fundamentally utilized in genomic profiling. Another study of six patients retaining metastatic PDAC validated the targeted scNGS method by exhibiting CTCs with SNVs regarding KRAS/TP53/SMAD4, in tandem with assuring positive immunofluorescent staining for Pan‐CK/vimentin/CD45 [100].

## 7 Current Status of NGS for Pancreatic Disease Diagnosis

The first report utilizing WES in familial PDAC detected a truncating mutation concerning the specific PALB2 gene, whereas both WES and WGS detected ATM gene mutations whose deleterious mutations were specified by Sanger sequence investigation for familial PDAC [101, 102]. In addition, mutational status regarding CDKN2A, TP53, KRAS, and SMAD4 driver genes were reviewed by high‐density SNP microarrays in tandem with WGS, WES, and RNA‐Seq to compare gene expression regarding familial and sporadic PDAC [103]. Furthermore, WGS and WES of specimens with familial PDAC unveiled 16 genes retaining greater than three private heterozygous truncating premature variants for the detection of germline mutations [104, 105]. The recently targeted sequencing encircling all coding regions recognized six genes incorporating CDKN2A, TP53, MLH1, BRCA2, ATM, and BRCA1 particularly associated with PDAC carrying a family history of PDAC and sporadic disease in 7.9% and 5.2%, respectively [106].

The assortments present in KRAS/GNAS mutations, in tandem with alterations prevailing in TP53/PIK3CA/PTEN, exhibited 89% and 100% sensitivity and specificity, respectively, for advanced neoplasia, according to NGS findings [107]. The high sensitivity of KRAS/GNAS mutations in conjunction with specificity regarding IPMNs (intraductal papillary mucinous neoplasms) and mucinous PCs (pancreatic cysts) respectively is noticed during NGS strategy regarding PCF contrasting Sanger sequencing [108]. Another recent study confirmed that targeted NGS with high sensitivity detects genes incorporating NRAS, HRAS, BRAF, TP53, PIK3CA, PTEN, KRAS, GNAS, AKT1, and CTNNB1 that are typically deleted and/or mutated in PCs along with advanced neoplasia [109]. Moreover, 100% and 96% sensitivity and specificity concerning an IPMN were observed during detection regarding KRAS and/or GNAS mutations, whereas 89% and 100% sensitivity and specificity concerning both an IPMN and MCNs (mucinous cystic neoplasms) regarding KRAS and/or GNAS mutations, were noticed during conducting NGS [110, 111]. The lowest limit of detection for mutant alleles for Sanger sequencing and NGS, respectively, is about 10%–20% and 3%–5%, demonstrating the sensitivity of NGS to detect a mucinous PC along with high‐ranking dysplasia in tandem with invasive adenocarcinoma. In contrast, NGS detected 49% mutations in KRAS and/or GNAS while Sanger sequencing detected 39% [112].

As an approach for the PCF detection abbreviated from pancreatic cyst fluid, NGS has recognized KRAS mutations providing 96%–100% specificity and 76%–89% sensitivity for MCNs and BD‐IPMNs [108, 109]. However, GNAS‐related mutations are not noted in MCNs but exhibited high specificity for IPMNs along with a 30%–45% prevalence in BD‐IPMNs [107, 113]. In addition, mutations regarding PIK3CA, PTEN, TP53, SMAD4, and/or AKT1 provided 96%–100% and 32%–79% specificities and sensitivities, respectively [32, 34, 47, 114, 115]. In addition, preoperative diagnosis regarding pancreatic cysts with supplemental value is apparent by dint of NGS through the recognition of 119 variants in 59% PCFs typical in cancer or IPMNs abbreviated from intraductal papillary mucinous neoplasms contrasting in non‐mucinous cysts [116]. Likewise, confirmation regarding a mucinous etiology in 72% of cysts and demonstration of a VHL mutation in tandem with TP53, GNAS, and KRAS mutations along with loss of CDKN2A, and SMAD4, is feasible through the NGS approach that validates the detection with reasonable read coverage concerning indels, in tandem with single nucleotide variants with allelic frequencies [32].

## 8 Limitations Along With Future Prospects of NGS for Pancreatic Disease Diagnosis

Although NGS is capable of detecting low‐abundance somatic mutations, the rate of sequencing errors due to NGS assays poses a challenge because at least more than 1% of sequence variants must be present for the mutations to be considered legitimate as opposed to being merely background sequencing errors [117, 118]. Basically, the low (0.1%–1%) somatic mutation concentrations in pancreatic juice in PDAC patients necessitate modified standard NGS protocols like “SafeSeq”‐like molecular strategies to distinguish actual low‐abundance somatic mutations even from low‐level errors that can be improved by digital NGS method, equivalent to digital PCR [119]. In a recent study, digital NGS was used to identify mutations in duodenal clusters of pancreatic juice to assess the diagnostic accuracy of this test in a group of patients with and without pancreatic ductal neoplasia. However, the highly specific digital NGS testing for pancreatic juice investigation is limited by the lack of sensitivity for a definitive pancreatic cancer diagnosis [120].

The enormous clinical use of NGS in PCF detection, which is unlocked by the reagent price control and batch specimen's capacity to increase NGS availability, is nonetheless constrained by relatively high costs; the current cost is one‐third comparable to an MRCP or MRI scan [114]. Therefore, in order to fully utilize the immense potential of NGS for diagnosing cases of pancreatic disease, different investigation, false‐positive results, perseverance of criteria, maintenance of unruffled probands, operating quality, quality governance, and ethical complaints instruct to be supported [121, 122].

## 9 Conclusion

Differentiating and identifying a wide range of clinical manifestations of pancreatic illness caused by specific genetic mutations might be difficult due to the growing number of suspected genes and intricate data analysis. The purpose of this review is to highlight several NGS approaches as a potential substitute for conventional methods in the diagnosis of pancreatic cancer and associated diseases. CT, MRI, contrast‐enhanced Doppler US, EUS, ERCP, transabdominal US, laparoscopy, and PET are a few instances of conventional diagnostic techniques with respective drawbacks. Genome analysis, including Sanger sequencing and NGS, is currently employed in the majority of diagnoses involving pancreatic disease. Millions to billions of tiny DNA fragments can be sequenced using the NGS techniques, which enables rapid sample screening with low‐abundance detection (0.1%–1% mutation prevalence), minimizing associated expenses. A variety of distinctive genes that have been linked to pancreatic illnesses can be managed to recognize utilizing the NGS technique, which also enables prompt, affordable, and accurate mutation identification on a comprehensive scale. Consequently, NGS has broadened the scope of genomes for pancreatic disease diagnosis, and compared to conventional diagnostic techniques, a number of advancements are now achievable through a range of strategies.

## Conflicts of Interest

The authors declare no conflicts of interest.

## Data Availability

Interested researchers can obtain the datasets used in this study by contacting the corresponding author.
